# Poverty Reduction Effects of Medical Insurance on Middle-Aged and Elderly Families under the Goal of Common Prosperity in China

**DOI:** 10.3390/healthcare11040477

**Published:** 2023-02-07

**Authors:** Linhong Chen, Xiaocang Xu

**Affiliations:** 1 School of Marxism, Chongqing Technology and Business University, Chongqing 400067, China; 2 School of Economics and Management, Huzhou University, Huzhou 313000, China

**Keywords:** common prosperity, poverty reduction effect, medical insurance, relative index, family medical financial burden

## Abstract

Eliminating poverty due to illness is an important way for China to pursue common prosperity. The high medical expenditure caused by the aging population has brought severe challenges to governments and families of all countries, especially in China, where the entire population has just been lifted out of poverty in 2020 and then hit by COVID-19. How to prevent the possible return of poor boundary families to poverty in China has become a difficult research topic. Based on the latest data from the China Health and Retirement Longitudinal Survey, this paper discusses the poverty reduction effect of medical insurance on middle-aged and elderly families from the absolute index and relative index. Medical insurance had a poverty reduction effect on middle-aged and elderly families, especially the poor boundary families. For example, people who participated in medical insurance reduced their financial burden by 2.36% for middle-aged and older families compared to people who did not participate in medical insurance. Furthermore, the poverty reduction effect had heterogeneity in gender and age. This research brings some policy implications. For example, the government should give more protection to vulnerable groups such as the elderly and low-income families and improve the fairness and effectiveness of the medical insurance system.

## 1. Introduction

**Figure 1:**
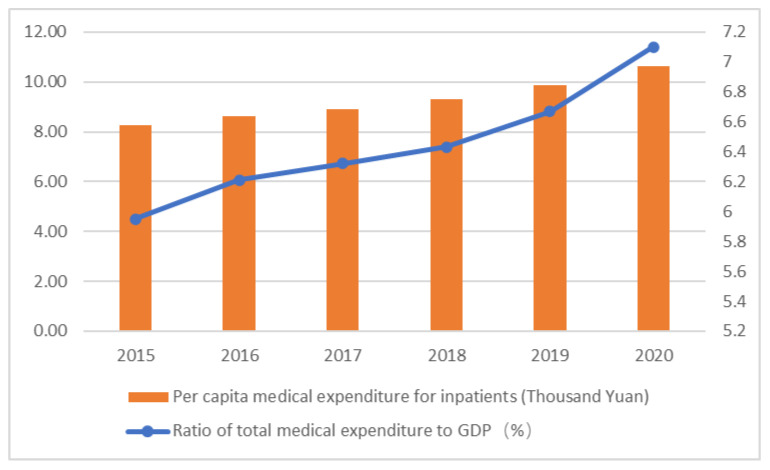
Burden of medical expenditure in China from 2015 to 2020. Source: China Health Statistical Yearbook 2021.

China has entered a stage of population aging, with the proportion of people aged 65 and above in the total population rising from 10.5% in 2015 to 13.5% in 2020. The dependency ratio of the elderly has also soared from 37% in 2015 to 45.9% in 2020, and about 190 million of them suffer from chronic diseases. This poses a serious challenge to both the government and families’ medical expenditure burden (China Health Statistical Yearbook, 2021). From 2015 to 2020, the proportion of total medical expenditure in GDP increased from 5.95% to 7.1%, the per capita medical expenditure for outpatients increased from CNY 233.9 to CNY 324.4, and the per capita medical expenditure for inpatients increased from CNY 8268.1 to CNY 10,619.2 (Figure 1). The rapid growth of medical expenditure puts a heavy economic burden on the whole of society [1]. At the same time, since the establishment of the basic medical insurance system in 1998, China has formed a basic pattern of social medical insurance, with the main insurance as the primary and commercial medical insurance as the auxiliary, but there are still some limitations such as system decentralization and system fairness to be improved. In addition, moral hazards, information asymmetry, and other issues, as well as public health emergencies such as COVID-19, have impacted the development of the health service system in China.

Scholars generally believe that medical insurance can cause the growth of the utilization rate of health services to varying degrees and improve the health status of residents [2,3]. The research on the impact of medical insurance on medical expenditure mainly focuses on the following four aspects.

First, medical insurance can improve the level of medical consumption and reduce the medical economic burden. Medical insurance subsidizes residents’ medical expenditures so that residents can enjoy the same or even better health services with lower out-of-pocket medical expenses, which can reduce the burden of residents’ medical expenditures. Zang and Zhang (2019) [4] believe that there is a substitution effect between medical insurance and family intergenerational economic support. Jung and Liu (2015) [5] and Kang and Barner (2017) [6] obtained two different opinions on the provision of medical insurance, i.e., the level of out-of-pocket medical expenditures decreases and there is no difference, respectively, while Choi et al. (2018) [7] and Feng et al. (2020) [8] confirmed the positive role of long-term care insurance. Furthermore, the higher the level of medical insurance, the more obvious its positive effect on medical expenditure [9,10].

Second, medical insurance can reduce the incidence of catastrophic medical expenditures and crowd out preventive saving behavior, which further effectively prevents the generation of “poverty caused by illness” [11,12]. Zhu and Shi (2016) [13] used heckprobit models to empirically study the impact of floating population participation in basic medical insurance on catastrophic medical expenditures and proposed that medical insurance with a higher reimbursement ratio of floating population participation is less prone to catastrophic medical expenditures.

Third, medical insurance shows heterogeneity in different income groups and the fairness of the system needs to be improved. Specifically, the medical expenditure effect of medical insurance on low-income groups is not obvious, but it can promote the medical expenditure of middle and high-income groups [14,15]. Yu et al. (2018) [16] found that the social medical insurance system did not have a significant positive impact on the medical consumption and health status of vulnerable elderly groups whereas Chen et al. (2019) [17] and Kwon et al. (2015) [18] found that medical insurance did not significantly reduce the medical expenditure of ordinary people and that the out-of-pocket medical expenditures of the rich are actually reduced more. 

Fourth, the positive effect of medical insurance on medical expenditure is limited by information asymmetry, moral hazard, and other factors [19,20]. Similarly, as medical insurance lowers the threshold of access to health services, residents can enjoy health services at a lower cost, which to a certain extent leads to the abuse of health services such as excessive medical treatment, resulting in the unreasonable growth of medical expenditure [21]. Ren et al. (2014) [22] and Zhao et al. (2021) [23] found that elderly people with poor health status were more inclined to participate in medical insurance, and that the frequency of participating in physical exercise decreased significantly after obtaining insurance, which confirmed the existence of adverse selection and moral hazard in medical insurance. 

In summary, the academia on the poverty reduction effect of medical insurance on elderly families has reached a basic consensus: medical insurance can reduce the medical expenditure burden of the government and the family and reduce the incidence rate of catastrophic health expenditure, which reduces the risk of family poverty due to illness. However, the actual effect of the poverty reduction effect is debated, due to imbalanced outpatient-hospitalization problems caused by information asymmetry and moral hazard, and the heterogeneity between different genders and ages and especially between high-income and low-income families. These problems reserve certain research space and hold value in this paper. Therefore, this paper raises the research question, what are the characteristics of the poverty reduction effect of medical insurance on middle-aged and elderly families under the background of China’s special national conditions? In order to answer this question, this paper selects the latest data from the China Health and Retirement Longitudinal Survey (CHARLS) and uses the absolute index (total medical expenditure) and relative index (proportion of medical expenditure in per capita annual income) to discuss the poverty reduction effect of medical insurance on middle-aged and elderly families and its characteristics regarding urban-rural, gender, and age differences.

## 2. Materials and Methods

### 2.1. Theoretical Analysis

The poverty reduction effect of medical insurance on middle-aged and elderly families is mainly through influencing individual health and the utilization of health services.

(1) Individual health. Medical insurance affects individual health status and medical expenditure through daily health behavior factors. On the one hand, medical insurance can remind residents to pay attention to their health status and improve their awareness of health care. On the other hand, the existence of medical insurance can reduce the economic cost of bad health behaviors to a certain extent. Moral hazards may encourage more bad health behaviors and bring a negative impact on residents’ health status. Cao and Tan (2022) [24] used the CHARLS data to empirically conclude that the frequency of physical examination of middle-aged and elderly people participating in medical insurance has decreased, which makes the health effect of medical insurance discounted due to moral hazard.
(2) Use of health services. Medical insurance reduces the relative price and access threshold of health services through the subsidy of medical expenditures, releasing the potential medical demand that had been restrained by economic factors, giving citizens more opportunities to improve their health status through health services, and at the same time increasing the consumption level of health services. Xu and Gu (2022) [25] used the differential-difference model and found that the critical illness insurance system could increase the hospitalization probability of middle-aged and elderly residents by 1.03%.

### 2.2. Variable Selection

Based on the above theoretical basis and research assumptions, this paper constructed the following variable system and analyzed the poverty reduction effect of medical insurance on middle-aged and elderly families by discussing the impact of medical insurance on their health status and medical expenditure.

#### 2.2.1. Explained Variables: Total Medical Expenditure (Absolute Index) and Family Medical Economic Pressure (Relative Index)

Most existing research tends to assess the utilization of health services from the use frequency and consumption expenditure of health services, such as outpatient expenditure, hospitalization expenditure, or total medical expenditure, within a certain period. This paper selects medical expenditure and medical economic burden for the family as explained variables. The total medical expenditure (absolute index) is the sum of the recent outpatients’ outlays and inpatients’ outlays in the past year, and the family medical economic burden (relative index) is the proportion of medical expenditure in the per capita annual income of middle-aged and elderly families.

#### 2.2.2. Main Explanatory Variable: Medical Insurance

Whether to participate in medical insurance (including medical insurance for urban and rural residents, medical insurance for urban residents, and new rural cooperative medical insurance) is selected as the core explanatory variable. In our paper, if the respondent has participated in medical insurance, the value assigned is 1, otherwise, the value assigned is 0. The corresponding title for the questionnaire is “Do you currently participate in the following medical insurance?”.

#### 2.2.3. Other Explanatory Variables

Medical expenditure is not only affected by the characteristics of medical insurance but is also affected by many other factors, including health status. When selecting other explanatory variables, this study selected other explanatory variables from four aspects: environmental factors, personal characteristics, health behaviors, and health outcomes. Take the nominal variables as an example: 1 for males and 0 for females in the Gender value; unmarried (never married or cohabitation) or divorced (no longer married, separated as a spouse, divorced, or widowed) is “0”, and married is “1” in the Marital status value, and so on. Additionally, in terms of the Ordinal variables, the self-rated health status was given as “0” for “very poor” and “poor”, “1” for “fair”, and “2” for “good” and “very good”. Weekly activity level was assigned with inactive as “0”, low-intensity physical activity as “1”, moderate-intensity physical activity as “2”, and high-intensity physical activity as “3”.

### 2.3. Empirical Tools

The medical expenditure data in CHARLS presented an obviously skewed distribution. If OLS regression is performed directly, it often leads to selective bias and other endogenous problems. Therefore, the two-part model and Heckman sample selection model are used in this paper to explore the impact of medical insurance on the medical expenditure of middle-aged and elderly people. First, the logarithm of medical expenditure data is processed, and then the two-part model and sample Heckman selection model are chosen, to deal with possible sample selection bias and selective bias problem, respectively, at the same time to test each other between the two models to determine if their conclusions are consistent; the empirical results are robust.

Firstly, the two-part model is used to deal with endogeneity problems caused by medical expenditure with a large number of “0” values. According to the model, the medical expenditure of micro-individuals is determined by the probability model of medical treatment (the probit model is usually used to estimate) and the medical expenditure level model (the log formalized least square method, LOLS), and the two models are independent of each other, that is, the level of individual medical expenditure has nothing to do with whether they choose medical treatment or not.

Secondly, the Heckman sample selection model is mainly used to solve the problem that the authenticity and reliability of samples collected are reduced because the medical expenditures of middle-aged and elderly individuals are not incurred due to self-selection The model consists of two parts: the selection equation, which mainly estimates whether an individual chooses to seek medical care (as shown in Equation (1)), and the expenditure equation, which is a regression estimate of the actual medical expenditure (as shown in Equation (2)).

(1) $$P_{i}=\{\begin{array}{c} 1,\;x_{i}\beta_{1}+\varepsilon_{1i}>0\\ 0,\;x_{i}\beta_{1}+\varepsilon_{1i}\leq 0 \end{array}$$

(2) $$\log(y_{i}|p_{i}=1)=x_{i}\beta_{2}+\varepsilon_{2i}$$

When $P_{i}$ takes is “0”, it represents medical expenditure without medical treatment, while when $P_{i}$ is “1”, it represents medical expenditure with medical treatment.

Moreover, in order to deeply analyze the poverty reduction effect of medical insurance on middle-aged and elderly families, we use the Generalized Linear Model to carry out empirical research from two aspects: the absolute index (total medical expenditure) and the relative index (family medical financial burden). STATA 16 is used for all data processing in this paper.

### 2.4. Data Source

The research data of our paper comes from the 2018 tracking data released by the China Health and Retirement Longitudinal Survey (CHARLS) research group. CHARLS is a large-scale interdisciplinary research project hosted by the National Development Research Institute of Peking University. It collects high-quality micro-data of families and individuals of middle-aged and elderly people aged 45 and above in China over the years, covering more than 10,000 households in 150 counties and 450 villages in 28 provinces, to help scholars carry out interdisciplinary research on population aging and health issues. According to the public information of the research group, the updated tracking data will be released in 2023. By the end of 2022, the academic community will have produced more than 4000 academic achievements using the free and open database of the research group. A total of 3599 valid samples were screened out in this study.

## 3. Results

This paper uses the absolute index (total medical expenditure) and relative index (family medical financial burden, namely the proportion of medical expenditure in per capita annual income of the family) as the evaluation carriers. Among them, the relative index is at the core of discussing the poverty reduction effect.

### 3.1. Statistical Descriptive Analysis

Table 1 shows the descriptive statistics of the sample.

As can be seen from Table 1, the self-rated health status of the mean is 0.597, which shows that most of the elderly’s subjective health status is poor; objective health also has the same conclusion, chronic illness conditions in older adults indicate an average of 1.121 kinds of chronic diseases. Thus, the necessity for “healthy aging” in China is urgent. The average medical expenditure of the middle-aged and elderly is CNY 7659.466, which may be affected by special circumstances, and the average total medical expenditure is relatively high. The mean value of medical insurance participation is 0.815, indicating that the vast majority of those sampled participate in some kind of medical insurance at present, which is also consistent with the medical insurance participation situation published in China’s current macro data. Sample description results in other explanatory variables show that the average age of the respondents is 63.807 years old, and most of them are rural residents with little education above primary school. The majority of those sampled are married, and the average annual household income is CNY 32,292.177. Most middle-aged and elderly people have no barriers to daily activities and they engage in moderate to high-intensity physical activity every week.

### 3.2. Absolute Index: Total Medical Expenditure

The empirical results are shown in Table 2.

First of all, after controlling other variables, the impact of medical insurance on the medical expenditure of middle-aged and elderly people is mainly reflected in the expenditure model, while the probability of seeing a doctor selection equation shows that the influence coefficient of participation in medical insurance on the probability of medical treatment of the elderly is positive, but this result is not significant. The results of the expenditure equation show that the total medical expenditure of middle-aged and old people who have participated in medical insurance is significantly lower than that of middle-aged and old people who have not participated in medical insurance. The two-part model shows that participation in medical insurance will reduce the total medical expenditure by 25.9% at a significance level of 5%. The Heckman sample selection model shows a 29.0% reduction in total health spending, and the results are significant at the 1% level.

Secondly, there are other influencing factors. The medical expenditure level of middle-aged and elderly male groups is higher than that of female groups. The two-part model shows that the total medical expenditure of the male group is 36.0% higher than that of the female group, and the medical expenditure of males in the sample selection model is 37.9% higher than that of females. For each additional year of age, the total health spending increases by about 3%. In addition, there is no significant association between total health expenditure and educational level. Moderate exercise is beneficial in reducing the total medical expenditure of middle-aged and elderly people. Compared with the inactive middle-aged and elderly people, the medical expenditure of those who participate in moderate-intensity physical activity per week is reduced by more than 40%, and the medical expenditure of those who participate in high-intensity physical activity is reduced by 70% at a significance level of 1%.

In addition, medical expenditure and self-rated health status show a coincident change relationship. The higher the self-rated health, the lower the medical expenditure. People with average self-rated health spend about 37% less than those with poor self-rated health, and those with good self-rated health spend more than 50% less than those with poor self-rated health, which is significant at the 1% level. For each additional chronic illness, the two models show a 13.4% increase in total medical expenditure, and the Heckman sample selection model shows a 12.5% increase in total medical expenditure.

Therefore, the empirical results of the two-part model are basically consistent with the Heckman sample selection model, indicating that the empirical results are robust.

### 3.3. Relative Index: Family Medical Financial Burden

This section uses the relative index of the proportion of medical expenditure in the annual per capita income of families and evaluates the negative effect of medical insurance on the medical economic pressure of middle-aged and elderly families based on the characteristics of the index data combined with the generalized linear model. The regression results are shown in Table 3.

First of all, taking the group not participating in medical insurance as the control group, participating in medical insurance helps the middle-aged and elderly families reduce their medical financial burden by 3.47%, which is significant at the 5% significance level. Participation in medical insurance is beneficial in relieving the medical and economic pressure on middle-aged and elderly families.

Secondly, there are other influencing factors. The medical and financial pressure on middle-aged and elderly men is 4.17% higher than that of women (*p* = 0.000). With the increase in age, the medical financial burden of middle-aged and elderly families increases by 0.11%. The older the family is, the higher the medical expenditure is, and the heavier the medical financial burden is on the family. The coefficient of influence of the accessibility of medical resources on a family’s medical financial burden is close to zero. The increase in family annual income is beneficial to reducing the medical financial burden of middle-aged and elderly families. In addition, the intensity of weekly physical activity has an impact on the family’s medical financial burden. For example, participation in low-, moderate-, and high-intensity weekly physical activity is associated with a reduction of 3.96% (*p* = 0.016), 4.97% (*p* = 0.003), and 9.72% (*p* = 0.000) in family medical financial burden, respectively, compared with no physical activity.

Third, the better the health status of the middle-aged and elderly, the lower the medical financial burden of their families. For each additional chronic disease, the financial burden of family health care increases by 1.31% (at the 1% significance level). The family medical financial burden of the middle-aged and old people with average self-rated health status is 3.36% lower than that of the middle-aged and old people with poor self-rated health status, and the family medical financial burden of the middle-aged and old people with poor self-rated health status is 5.81% lower.

### 3.4. Heterogeneity Analysis: Gender and Age

To test the heterogeneity of the impact of medical insurance proposed in some of the existing research results, this paper adopts the sub-sample regression method to conduct a classification regression according to the gender and age of the samples and to explore the group differences in the impact of medical insurance. The results are shown in Table 4.

First, gender differences are examined. Participation in medical insurance reduces the total medical expenditure of middle-aged and elderly females by 24.0% and that of middle-aged and elderly males by 26.1%, at a significance level of 10%, compared with the group that has not participated in medical insurance. This gender difference is also reflected in the impact on family medical burden. Participation in medical insurance reduces the family medical financial burden of a middle-aged and elderly male by 3.3%, this result is significant at the level of 10%, but the effect on the family medical financial burden of a middle-aged and elderly female is not significant. Therefore, the negative effect of medical insurance on the total medical expenditure of middle-aged and elderly males is slightly stronger than that of middle-aged and elderly females, and the negative effect on the family medical financial burden of middle-aged and elderly males is also stronger than that of females. 

Second, the influence of age differences is explored. Participation in medical insurance has no significant effect on the 45–59 age group, but it can promote their utilization of health services for the elderly group aged 60 and above. It reduces their medical expenditure by 37.4% and their family medical economic burden by 4.7% at the significance level of 1%. This shows that the effect of medical insurance is more significant for the elderly group over 60 years.

## 4. Discussion

A high medical financial burden constitutes an important reason for some families to return to poverty, therefore, medical insurance plays an important role in preventing families from returning to poverty. This paper selected the middle-aged and elderly with a high demand for health services as the research object and the microdata of the latest CHARLS as the data source. Moreover, the poverty reduction effect of medical insurance on middle-aged and elderly families from the perspectives of the absolute index (total medical expenditure) and relative index (proportion of medical expenditure in per capita annual income) were discussed; some meaningful arguments were found.

First, there is a substitution effect between medical insurance and total medical expenditure. Similar to the research by Zang and Zhang (2019) [4] and Liu (2015) [9], medical insurance was found to be beneficial in significantly reducing the total medical expenditure of middle-aged and elderly people. The total medical expenditures of people with medical insurance were significantly lower than those of people who did not participate in medical insurance. In addition, the two-part model showed that participation in medical insurance reduced the total medical expenditure by 25.9% at a significance level of 5%. The Heckman sample selection model showed that participation in medical insurance resulted in a 29.0% reduction in total medical expenditure at a significance level of 1%. Two models were selected at the same time to better avoid the problem of sample endogeneity, and the empirical results obtained by the two empirical models were the same, indicating that the empirical results are relatively robust.

Second, medical insurance had a negative effect on middle-aged and elderly families. Medical insurance reduced the medical burden of middle-aged and elderly families. Compared with people who did not participate in medical insurance, participating in medical insurance helped them to reduce 2.36% of the medical financial burden at a 10% significance level, indicating that medical insurance may be beneficial for people by reducing medical expenditure by reducing precautionary savings. Although there are some differences in specific data, this further confirms the arguments of Ding and You (2019) [12] and Zhu and Shi (2016) [13].

Thirdly, the poverty reduction effect of medical insurance on middle-aged and elderly families had heterogeneity in gender and age. Compared with females, the negative effect of medical insurance was more significant for males. Participation in medical insurance reduced the total medical expenditure of middle-aged and elderly males more than that of middle-aged and elderly females. Medical insurance reduced the family medical burden of middle-aged and elderly males by 3.3% at a significance level of 10%, while the effect on the family medical burden of middle-aged and elderly females was not obvious.

Finally, appropriate participation in sports was beneficial for reducing the pressure of medical expenditures for the middle-aged and elderly. Compared with the middle-aged and elderly people who did not exercise, the total medical expenditure and family medical financial pressure of the middle-aged and elderly people who did exercise moderately every week was lower. In addition, taking part in low-intensity physical activity every week reduced the family’s medical financial burden by 4.06% (*p* = 0.004), taking part in moderate-intensity physical activity every week reduced it by 6.03% (*p* = 0.000), and participation in high-intensity physical activity every week reduced it by 9.54% (*p* = 0.000). Therefore, our conclusion is the same as Ren et al. (2014) [22]: participation in physical activity significantly reduced the pressure of medical expenditure on middle-aged and elderly people by reducing their probability of illness.

However, there are inevitably some study limitations. For example, a longitudinal comparative study on the historical data of CHARLS (2008, 2011, 2015, and 2018) was not conducted; perhaps the longitudinal research results of the multi-year data are more accurate. In addition, our research data is taken from the 2018 data, before the outbreak of COVID-19 which has had a huge impact on China’s national economy and households over the past three years and may also cause some deviation from our research conclusions. 

Nevertheless, our research still brings some policy implications. First, the government should further expand medical insurance coverage to ease financial burden and promote equal access to health. For example, the government should strengthen the publicity of the important role of medical insurance, especially for middle-aged and elderly individuals with poor health, and there should be a large demand for medical services to participate more in medical insurance so that they can enjoy the welfare policies of medical insurance, improve the overall health level of the people, and relieve the pressure of rising medical expenses. Secondly, the government should give more protection to vulnerable groups, such as the elderly and low-income families, and improve the fairness and effectiveness of the medical insurance system. The effect of medical insurance on the medical expenditure of middle-aged and elderly families is more significant than in urban and high-income groups. Therefore, the marginal utility of medical insurance for the middle-aged and elderly and low-income groups is higher. From the perspective of improving the fairness and efficiency of the system, medical insurance should be strengthened in the protection of vulnerable insured groups by expanding the coverage of low-income groups, reducing the proportion of self-payment and other measures, and providing more welfare policies to middle-aged and elderly people and low-income groups. Finally, the government should improve the supporting mechanism of medical insurance and the medical security system. Although medical insurance reduces the medical burden of the insured object, the effect is limited. With the rapid increase in the care and medical needs of the disabled elderly, it is urgent to introduce corresponding countermeasures, such as the promotion of long-term care insurance policies, to effectively disperse the pressure faced by medical insurance.

## 5. Conclusions

Our research results further verify that medical insurance can significantly reduce the total medical expenditure of middle-aged and elderly people, thus reducing the medical burden of middle-aged and elderly families. That is, medical insurance has a poverty reduction effect on middle-aged and elderly families, and there is heterogeneity in gender and age. Accordingly, we propose to further expand the scope of medical insurance to provide more protection for the elderly and low-income families, as well as other vulnerable groups, and improve the fairness and effectiveness of the medical insurance system. However, the pressure of government financial expenditure (especially under the impact of continuous COVID-19), the existence of a moral hazard, and other factors have reduced the effectiveness of medical insurance. The long-term care insurance system, which is relatively mature in Germany, Japan, and other developed countries but has only begun to pilot in China in 2016, can play a certain auxiliary role. 

Future academic research can be further expanded in the following aspects: first, integrate the latest CHARLS data with previous data to explore the changing process of the poverty reduction effect of medical insurance on middle-aged and elderly families. Second, focus on adverse selection and moral hazard in medical insurance and evaluate the impact of adverse selection and moral hazard on the release effect and negative effect of medical insurance demand. Third, evaluate the tacit degree and actual effectiveness of various medical insurance divisions and cooperations, combined with other medical insurance systems in China, and explore the overall improvement plan of the current medical insurance system in China, including further improvement of the auxiliary role of the long-term nursing insurance system.

## Figures and Tables

**Table 1:** Sample descriptive statistics.

Variable	Sample Size	Mean	Variance	Minimum	Maximum
Total medical expenditure	3599	7659.466	29,783.462	0	1,402,800
Family medical financial burden	3599	2.084	16.119	0	600
Number of chronic diseases	3599	1.121	1.258	0	9
Age	3599	63.807	9.972	45	95
Access to medical resources	3599	30.696	145.117	0	6000
Total annual household income	3599	32,292.177	75,975.197	2	1,930,000

**Table 2:** The substitution effect of medical insurance on total medical expenditure.

Variable	Two-Part Model		Heckman Sample Selection Model	
	Probability Model of Medical Treatment	Medical Expenditure Level Model	The Selection Equation	The Expenditure Equation
Medical insurance (uninsured as control group)	0.246	−0.259 **	0.168	−0.290 ***
	(0.184)	(0.101)	(0.177)	(0.103)
Male (female as control group)	−0.219	0.360 ***	−0.285 **	0.379 ***
	(0.142)	(0.0719)	(0.121)	(0.0732)
Age	−0.012	0.0258 ***	−0.018 **	0.027 ***
	(0.007)	(0.004)	(0.008)	(0.004)
Education level (no education as control group)				
Primary and below	0.240	−0.106	0.0778	−0.140
	(0.152)	(0.085)	(0.148)	(0.087)
Above primary school, secondary school, and below	0.505 **	0.086	0.125	0.0281
	(0.220)	(0.104)	(0.224)	(0.106)
Junior college or above	−0.052	−0.406	0.073	−0.374
	(0.381)	(0.275)	(0.301)	(0.278)
Married (single as control group)	0.253 *	0.204 **	0.130	0.165 *
	(0.152)	(0.094)	(0.131)	(0.095)
Access to medical resources	0.006	0.002 ***	0.011***	0.002 ***
	(0.004)	(0.000)	(0.004)	(0.000)
Log (total annual household income)	0.055	0.007	0.058	0.000
	(0.045)	(0.023)	(0.042)	(0.024)
Weekly physical activity (no exercise as control group)				
Low intensity	−0.183	−0.269 **	0.0435	−0.244 **
	(0.242)	(0.114)	(0.224)	(0.116)
Moderate intensity	−0.376	−0.462 ***	−0.146	−0.416 ***
	(0.247)	(0.117)	(0.227)	(0.119)
High intensity	−0.285	−0.737 ***	−0.088	−0.699 ***
	(0.258)	(0.121)	(0.237)	(0.123)
Self-rated health status (poor as control group)				
Fair	−0.017	−0.376 ***	−0.084	−0.375 ***
	(0.141)	(0.071)	(0.122)	(0.073)
Good	−0.279	−0.583 ***	−0.079	−0.539 ***
	(0.195)	(0.119)	(0.164)	(0.121)
Number of chronic diseases	0.088	0.134 ***	0.087	0.125 ***
	(0.060)	(0.027)	(0.054)	(0.027)
Constant	2.223 ***	6.237 ***	2.518 ***	6.306 ***
	(0.812)	(0.426)	(0.839)	(0.435)
Sample size	3599	3599	3599	3599

Note: * *p* < 0.1, ** *p* < 0.05, *** *p* < 0.01. Standard errors are in parentheses.

**Table 3:** Research on the negative effect of medical insurance on the family medical financial burden.

Variable	Family Medical Financial Burden	Variable	Family Medical Financial Burden
Medical insurance (uninsured as control group)	−0.0347 **	Married (single as control group)	−0.0002
	(0.0145)		(0.0135)
Male (female as control group)	0.0417 ***	Access to medical resources	0.0002 ***
	(0.0103)		(0.0000)
Age	0.0011 *	Log (Total annual household income)	−0.1108 ***
	(0.0006)		(0.0034)
Education level (no education as control group)		Weekly physical activity (No exercise control group)	
Primary and below	−0.0108	Low intensity	−0.0396 **
	(0.0123)		(0.0164)
Above primary school, secondary school, and below	0.0034	Moderate intensity	−0.0497 ***
	(0.0149)		(0.0168)
Junior college or above	−0.0453	High intensity	−0.0972 ***
	(0.0395)		(0.0174)
Self-rated health status (poor as control group)		Number of chronic diseases	0.0131 ***
			(0.0038)
Fair	−0.0336 ***	Constant	1.9297 ***
	(0.0102)		(0.0614)
Good	−0.0581 ***		
	(0.0171)		

Note: * *p* < 0.1, ** *p* < 0.05, *** *p* < 0.01. Standard errors are in parentheses.

**Table 4:** Heterogeneity analysis (gender differences and age differences).

		Observations	Total Medical Expenditure		Family Medical Financial Burden
			Probit	LOLS	
Gender differences	Female	2109	0.307	−0.240 *	−0.029
			−0.271	−0.142	−0.022
	Male	1490	0.194	−0.261 *	−0.033 *
			−0.261	−0.145	−0.018
Age differences	Aged 45–59	1289	0.047	0.009	0.002
			−0.464	0.182	0.031
	Aged 60 and above	2310	0.398 *	−0.374 ***	−0.047 ***
			−0.217	−0.123	−0.016

Note: * *p* < 0.1, *** *p* < 0.01. Standard errors are in parentheses.

## Data Availability

Please refer to: http://charls.pku.edu.cn/index.html (accessed on 16 May 2021).
